# Beyond Depression: Suicidality As the First Clue to a Silent Brain Tumor in the Elderly

**DOI:** 10.7759/cureus.117812

**Published:** 2026-10-12

**Authors:** Uaiye Enosolease, Oladele D Owasoyo, Joshua Van Le, Arindam C Chakrabarty

**Affiliations:** 1 Psychiatry, Southern Illinois University School of Medicine, Springfield, USA

**Keywords:** acute suicidality, brain tumors, depression, elderly, neuroimaging, organic etiology, premature diagnostic closure

## Abstract

Depression can be difficult to identify in the elderly due to the high prevalence of comorbid medical conditions and underlying neurological pathology. Consequently, there is a risk of diagnostic overshadowing in patients with pre-existing psychiatric or cognitive disorders. This case reports a woman with intellectual disability and a longstanding history of depression who developed sudden-onset fleeting suicidal ideation, initially attributed to the acute onset of a depressive episode. The dramatic appearance of suicidal thoughts was accompanied by regression to a childlike demeanor and an incongruent affect, prompting neuroimaging workup that revealed a large, centrally necrotic mass in the right temporal lobe. This case underscores the diagnostic challenges in distinguishing primary psychiatric illness from neuropsychiatric presentations of brain tumors, which can remain neurologically silent and present early on with psychiatric symptoms. It calls the attention of all clinicians to the importance of maintaining a high index of suspicion for organic etiologies in older patients with atypical psychiatric presentations.

## Introduction

The Diagnostic and Statistical Manual of Mental Disorders, Fifth Edition, Text Revision (DSM-5-TR) specifies that a major depressive episode in major depressive disorder (MDD) requires five or more symptoms during the same two-week period, representing a change from previous functioning, including depressed mood or loss of interest or pleasure. Other symptoms include low energy, changes in weight or appetite, sleep disturbances, feelings of worthlessness or excessive guilt, difficulties in concentration, psychomotor changes, and recurrent thoughts of death. The symptoms must cause clinically significant distress or functional impairment [1]. The lifetime prevalence of depression is estimated to be approximately 20% [2]. In recent studies, the prevalence in older adults varies from 10%-20% and is frequently masked by cognitive deficits and comorbid anxiety symptoms [3]. A meta-analysis reported a pooled lifetime prevalence of suicide attempts of approximately 31% among individuals with MDD [4]. MDD has also been reported to account for up to 87% of suicide deaths [5].

Depression and consequent suicidal ideation in patients with brain tumors have a multifactorial etiology. These factors may be individual-related, tumor-related, and/or disease-related [6]. One explanation is that neurocognitive changes and psychiatric symptoms, such as depression and/or suicidality, may result from the tumor’s mass effect, cerebral edema, or tissue disruption. These psychiatric symptoms may precede overt neurological findings and serve as early indicators of intracranial pathology [7]. Rarely, brain tumors may present solely with psychiatric manifestations, necessitating a more robust diagnostic evaluation. The aim is to facilitate early detection, especially in patients with no prior psychiatric history, atypical presentations, and/or treatment resistance [8]. The incidence of primary malignant brain tumors is estimated at seven per 100,000 individuals and increases with age. About half of these tumors are glioblastomas, and frontal or temporal lobe tumors are more likely to present with neuropsychiatric symptoms [9].

The challenge therein lies in delineating primary psychiatric symptoms from those secondary to an intracranial space-occupying lesion. In clinical practice, it is common to have a high index of suspicion for organic etiologies in older adults presenting with new-onset psychiatric symptoms, resulting in a lower threshold for pursuing diagnostic investigations such as neuroimaging. Conversely, an established psychiatric history may contribute to premature diagnostic closure [8]. Suicide-risk assessment should nevertheless consider psychiatric symptoms and physical health conditions [10]. In this case, we report an elderly woman with a known history of MDD who presented with acute worsening of depressive symptoms and a first-time occurrence of active suicidal ideation. Despite the apparent similarities with her primary psychiatric diagnosis, the presence of subtle atypical features supported the need for further evaluation. This case highlights the importance of keeping a high index of suspicion for underlying brain pathology, particularly in older adults with atypical presentations. Neuropsychiatric manifestations may precede or conceal focal neurologic deficits, potentially obscuring the diagnosis, thereby adversely impacting outcomes.

## Case presentation

We present the case of a 69-year-old white female who was brought to the emergency department (ED) by her family members following referral by her psychiatrist for acute-onset suicidal ideations and auditory hallucinations. Although the exact IQ was unknown, the family reported a diagnosis of mild intellectual disability secondary to birth-related trauma as well as a psychiatric history of MDD. She endorsed a noticeable decline in mood, with intermittent mood fluctuations over the prior week and changes in her energy level; however, she denied having anhedonia. She reported decreased appetite due to food tasting bad in her mouth, as well as abdominal pain of four days’ duration. The patient endorsed poor sleep, averaging four hours nightly over the past week compared to her baseline of eight hours. She endorsed persistent worry and uneasiness as she often felt on edge. On evaluation, she denied any current suicidal or homicidal ideations and identified several reasons to live, including a desire to enjoy life and nature, especially flowers, and to watch her favorite sitcoms. She reported having thoughts of self-harm and death the night prior. Recent stressors included giving her pets away due to limited mobility from a leg fracture and the upcoming birthday of her deceased brother, although she minimized its significance as they were not close. Notably, the sudden emergence of suicidal thoughts was accompanied by a regression to a childlike demeanor and an affect incongruent with the situation, raising further concern about her mood regulation. This contrast between her recent suicidal ideation and later heightened affective state highlighted a concerning mood fluctuation and contributed to the decision to pursue further medical evaluation. Initially, the patient denied auditory or visual hallucinations; however, she later reported hearing a bell the previous night.

Collateral information from the patient’s nieces revealed that over the past week, she had not been her usual self, and there was a drastic change from her baseline behavior, beginning approximately five days prior. Despite her intellectual disability, they described her as typically independent, confident, and lively. Over the last week, she seemed confused and appeared to be hallucinating. She would claim that a family member was calling her, although that individual was not present at the time. The night prior to presentation, she contacted her family, sounding distraught and hysterical while expressing suicidal intent, including thoughts of deliberately driving her vehicle into a specific oncoming train. Evaluation at an urgent care clinic a few days earlier included routine laboratory tests and abdominal ultrasound, which revealed no apparent abnormalities. Both the patient and her family denied any prior episodes suggestive of manic or hypomanic symptoms and past hallucinations or paranoid delusions. They also reported some falls in recent weeks, which were not associated with any loss of consciousness or apparent neurologic sequelae. She had been maintained on fluoxetine for years, though the dose had recently been reduced from 60 mg to 50 mg by her psychiatrist approximately two weeks prior to presentation. The reasons for the dose titration were not clearly established. The patient’s healthcare power of attorney (HCPOA) requested psychiatric admission, expressing concerns that the patient posed an acute danger to herself and lacked the capacity for independent living and self-care.

The patient was observed during the interview as well as from a distance when she was unaware of the team. Her mood had initially been reported as “good,” but her affect was anxious as she clutched her blanket tightly. She repeatedly pleaded to go home, stating she did not wish to be in the hospital. Notably, there were rapid fluctuations in her affect, from anxious to cheerful and back to anxious again. Given the acute onset of suicidality, initial sad and anxious affect, and then the almost carefree affect, there was suspicion of mood lability. Cognitively, she was alert, oriented fully to person and place and partially to time, incorrectly identifying the day and month. Her attention and concentration were intact. However, there were some impairments in memory: she could recall only two out of three objects on short-term testing, and she struggled to recall basic biographical details. These findings were interpreted in the context of her pre-existing intellectual disability. On physical examination, her vital signs were as follows: temperature was 36.5°C, blood pressure 151/84 mmHg, pulse 89 beats per minute, and respiratory rate 18 breaths per minute. Her oxygen saturation was 94% on room air, and she was conscious with a Glasgow Coma Scale score of 15 [11]. Her face was symmetric, and no focal neurologic deficits were observed.

Investigations

The patient’s initial vital signs and laboratory findings are summarized in Table 1.

**Table 1:** Initial vital signs and laboratory findings Laboratory reference ranges are those reported by the performing institution; general adult reference values are shown for vital signs. The urine drug screen included amphetamines, barbiturates, benzodiazepines, cannabinoids, cocaine, propoxyphene, methadone, methaqualone, opiates, and phencyclidine. A hyphen indicates that no measurement unit applies. ALP, alkaline phosphatase; ALT, alanine aminotransferase; AST, aspartate aminotransferase; BUN, blood urea nitrogen; CO₂, carbon dioxide; HPF, high-power field; MCH, mean corpuscular hemoglobin; MCHC, mean corpuscular hemoglobin concentration; MCV, mean corpuscular volume; RBC, red blood cell; RDW, red cell distribution width; T3, triiodothyronine; T4, thyroxine; TSH, thyroid-stimulating hormone; WBC, white blood cell

Category	Test	Result	Unit	Reference range
Vital signs	Temperature	36.5	°C	36.0-37.5
	Blood pressure	151/84	mmHg	<120/<80
	Pulse	89	beats/min	60-100
	Respiratory rate	18	breaths/min	12-20
	Oxygen saturation, room air	94	%	95-100
Neurologic assessment	Glasgow Coma Scale score	15	points	15
Complete blood count	Hemoglobin	13.3	g/dL	12.0-16.0
	Hematocrit	40	%	37-47
	RBC count	4.92	×10⁶/µL	4.20-5.40
	MCV	81	fL	81-94
	MCH	27.0	pg	27.5-33.2
	MCHC	33.4	g/dL	31.0-36.0
	RDW	14.8	%	11.7-15.5
	WBC count	10.1	×10³/µL	3.4-9.4
	Absolute neutrophils	7.3	×10³/µL	1.8-6.5
	Absolute basophils	0.0	×10³/µL	0.0-0.2
	Absolute eosinophils	0.0	×10³/µL	0.0-0.4
	Absolute monocytes	0.9	×10³/µL	0.2-0.8
	Absolute lymphocytes	1.8	×10³/µL	0.9-3.0
	Neutrophils	72	%	47-67
	Eosinophils	0	%	0-6
	Basophils	1	%	0-2
	Monocytes	9	%	1-9
	Lymphocytes	18	%	25-45
	Platelets	237	×10³/µL	140-410
Chemistry	Sodium	142	mmol/L	136-145
	Potassium	2.7	mmol/L	3.5-5.1
	Chloride	101	mmol/L	98-107
	CO₂	30	mmol/L	21-31
	Anion gap	11	mmol/L	8-16
	BUN	17	mg/dL	7-25
	Creatinine	0.9	mg/dL	0.6-1.3
	Glucose	99	mg/dL	70-105
	Calcium	9.2	mg/dL	8.6-10.3
Liver tests	ALP	79	U/L	34-104
	AST	20	U/L	13-39
	ALT	31	U/L	7-52
	Total bilirubin	0.6	mg/dL	0.3-1.0
	Total protein	5.9	g/dL	6.0-8.3
	Albumin	4.0	g/dL	3.5-5.7
Thyroid studies	Free T3	2.5	pg/mL	2.4-4.4
	Free T4	1.3	ng/dL	0.5-1.3
	TSH	0.44	µIU/mL	0.45-5.33
Toxicology	Urine drug screen	Not detected	-	Not detected
	Urine ethanol screen	Not detected	-	Not detected
	Breath alcohol concentration	0.000	g/L	0.000-0.019
Urinalysis	pH	6.0	-	4.5-7.5
	Specific gravity	1.020	-	1.003-1.030
	Protein	30	mg/dL	Negative
	Glucose	Negative	-	Negative
	Ketones	Negative	-	Negative
	Bilirubin	Negative	-	Negative
	Hemoglobin	Negative	-	Negative
	Urobilinogen	≥4.0	mg/dL	Normal
	Nitrite	Negative	-	Negative
	Leukocyte esterase	Moderate	-	Negative
	WBCs	30	/HPF	0-4
	RBCs	1	/HPF	0-2
	Squamous epithelial cells	6	/HPF	0-5
	Bacteria	Few	-	None
Microbiology	Urine culture	Moderate probable contaminants	-	Not applicable

A comprehensive metabolic panel revealed moderate hypokalemia of 2.7 mmol/L. Other electrolytes and liver function enzymes were within the reference range. Urinalysis was significant for moderate leukocyte esterase and white blood cells. Urine culture later suggested these were probably contaminants. Thyroid-stimulating hormone was slightly low at 0.44 µIU/mL, but free T3 and T4 levels were normal. Complete blood count, urine toxicology screen, and breath ethanol test were also generally unremarkable. Given the patient’s atypical and acute presentation, combined with the recent history of falls, brain CT was recommended to rule out organic causes rather than admission to psychiatry. The patient’s hypokalemia was corrected in the ED. A non-contrast brain CT subsequently revealed a new mass-like lesion in the right temporal lobe, associated with adjacent edema. No acute hemorrhage was seen. The patient was educated on the findings and admitted to the medical service. Neurosurgery was consulted, and a detailed neurologic examination was performed, which revealed no abnormalities. Brain MRI with and without contrast confirmed a large, enhancing, centrally cystic and necrotic mass centered in the right temporal lobe, along with mild right uncal herniation (Figures 1A-1D). Electroencephalography showed no evidence of epileptiform discharges or seizure activity. A CT scan of the chest, abdomen, and pelvis, performed to evaluate for a possible primary malignancy or metastatic disease, revealed cardiomegaly, a multinodular goiter, a non-obstructing right renal stone, and colonic diverticulosis, but no evidence of a primary neoplasm or metastatic lesions.

**Figure 1:**
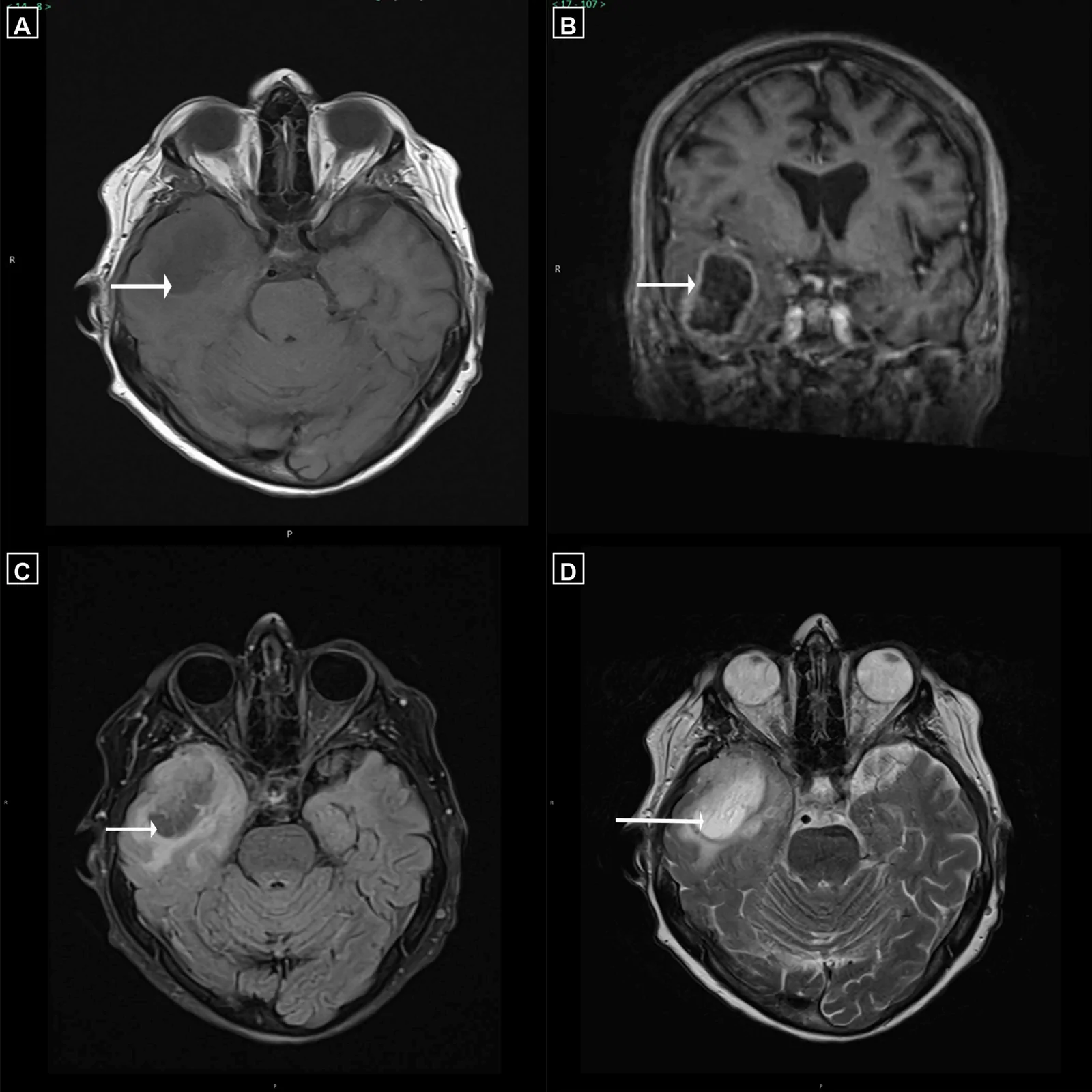
Brain MRI demonstrating the large, centrally cystic and necrotic mass with surrounding edema centered in the right temporal lobe (A) Axial precontrast T1-weighted image. (B) Coronal postcontrast T1-weighted image. (C) Axial FLAIR image. (D) Axial T2-weighted image. Arrows indicate the lesion. FLAIR, fluid-attenuated inversion recovery; MRI, magnetic resonance imaging

Differential diagnosis

The differential diagnosis included recurrent severe MDD because of the patient’s longstanding history; delirium due to another medical condition because of the transient suicidal ideation, auditory hallucinations, and reported confusion; and subdural hematoma because of the recent falls. Neuroimaging ultimately demonstrated a right temporal lobe tumor with adjacent vasogenic edema.

Outcome and follow-up

The patient and her family declined surgery and other interventions, and she was subsequently discharged to the care of her family and continued to follow up with her outpatient psychiatrist until she died two months later.

## Discussion

This case highlights the critical importance of maintaining a high index of suspicion for underlying organic pathology in elderly patients, even when there is a pre-existing psychiatric diagnosis. Acute psychiatric symptoms, particularly when atypical or inconsistent with a patient’s baseline, should not be prematurely attributed to known mental health disorders or age-related neurocognitive decline. In such scenarios, failure to pursue further evaluation, including neuroimaging when clinically indicated, may lead to delayed diagnosis and adversely affect patient outcomes [12]. This risk may be further amplified in individuals with prior psychiatric diagnoses, as clinicians may anchor on the existing psychiatric conditions while overlooking new or atypical features [8]. This is known as diagnostic overshadowing, which refers to a bias in which a patient’s symptoms are misattributed to a pre-existing psychiatric illness rather than considering a new diagnosis. A related cognitive bias, premature diagnostic closure, occurs when clinicians jump to a diagnosis too soon, failing to consider other explanations. Multiple factors, such as the patient’s longstanding psychiatric history, intellectual disability, recent psychosocial stressors, and a decrease in antidepressant dosage, complicated the interpretation of her atypical presentation, which was characterized by apparent regression in functioning. These factors increased the risk of making a premature primary psychiatric diagnosis, which may have resulted in admitting the patient to a psychiatric ward as requested by the HCPOA. Hence, a focused diagnostic workup is essential to avoid significant delays in proper diagnosis and management [13].

Comprehensive assessment in older individuals is important, and an ideal practice should encompass a thorough medical and neurologic workup, including neuroimaging, particularly in those presenting with rapid worsening of mood, cognition, or behavioral symptoms [8,14]. While our patient already had an established psychiatric history in conjunction with mild intellectual disability that may have explained her symptoms, the history from her family members suggested atypical features. They identified a clear acute deterioration from her stable baseline functioning, characterized by new behavioral changes and functional decline, highlighting the importance of collateral information. Collateral history from individuals who are familiar with the patient’s baseline functioning is critical for providing insights that may not be readily apparent to the evaluating clinician. In this case, extensive laboratory testing revealed hypokalemia, which was also considered, as electrolyte disturbances may contribute to neuropsychiatric symptoms. However, the severity and persistence of symptoms were not fully explained by this finding. The initial workup alone would not have led to the identification of the silent brain tumor. It is widely known that new-onset psychiatric symptoms in the elderly warrant an evaluation for potential organic etiologies [8].

It is not uncommon for central nervous system tumors to present with mood disturbances, behavioral changes, suicidal thoughts, or psychotic symptoms. The etiology of these symptoms may stem from direct tumor effects, vasogenic edema, or disruption of neural pathways, especially when located in silent brain lesions [7,15,16]. Notably, while the literature has disputed any association between psychiatric symptoms and tumor location or histological type [8], the later discovery of a brain tumor in our patient’s temporal lobe with imaging features suggestive of glioblastoma is consistent with reports that temporal lobe glioblastoma multiforme can sometimes present with psychiatric symptoms [17]. Despite a past psychiatric history of depression, the patient’s presentation with acute suicidality was a striking departure from her optimistic premorbid personality. Studies have revealed that patients with malignant brain tumors, particularly glioblastoma, have a markedly elevated risk of suicide, especially within the first year after diagnosis, and this has been found to be significantly higher in males, the elderly, and those with supratentorial tumors [18,19]. Subtle symptoms may contribute to missed opportunities for diagnosing brain tumors [20]. However, to the best of our knowledge, suicidality as a presenting feature of brain tumors appears to be rarely reported in the literature.

Although this patient declined further treatment after her diagnosis, discharging her prior to her tumor discovery could have resulted in worsening of her symptoms, repeated ED visits, and antidepressant treatment failure, with consequent worry and distress for both the patient and her family. Thus, missed diagnoses often result in increased morbidity and diagnostic delays, potentially worsening patient outcomes [20]. The extent to which tumor presence and identification influenced suicide risk cannot be determined from this case.

## Conclusions

Acute suicidality in elderly patients warrants a comprehensive diagnostic evaluation. This case underscores the need to maintain a reasonable threshold for considering organic causes in older patients with acute and/or atypical psychiatric presentations, including suicidality. Greater caution should be exercised, particularly in patients who already have a known psychiatric history, as organic etiologies may otherwise be overlooked in this population. Timely intervention, including neuroimaging when clinically indicated and multidisciplinary assessment, is key to minimizing misdiagnosis and optimizing outcomes.
